# Skeletal Muscle CSE Deficiency Leads to Insulin Resistance in Mice

**DOI:** 10.3390/antiox11112216

**Published:** 2022-11-09

**Authors:** Miaomiao Xu, Xiaoguang Liu, Peng Bao, Yanjie Wang, Xiaoyan Zhu, Yujian Liu, Xin Ni, Jianqiang Lu

**Affiliations:** 1 School of Exercise and Health, Shanghai University of Sport, Shanghai 200438, China; 2 School of Sport and Health, Guangzhou Sport University, Guangzhou 510500, China; 3 Department of Physiology, Navy Medical University, Shanghai 200433, China; 4 Research Center for Molecular Metabolomic, Xiangya Hospital Central South University, Changsha 410008, China

**Keywords:** H_2_S, cystathionine-γ-lyase, skeletal muscle, glucose tolerance, insulin resistance, exercise

## Abstract

Cystathionine-γ-lyase (CSE) is expressed in various tissues and generates H_2_S via an alternative desulfuration reaction. We sought to explore the functions of skeletal muscle CSE using skeletal muscle conditional knockout CSE (MCSEKO) mice. It was found that body weight, muscle morphology, and exercise capacity were not altered in MCSEKO mice compared with littermate wild-type mice. RNA-seq-based transcriptome analysis showed that 275 genes were differentially regulated in skeletal muscle and multiple signaling pathways including insulin signaling and mTOR, PI3K-AKT, and cGMP-PKG signaling pathways were enriched in MCSEKO mice. The intraperitoneal glucose tolerance test and insulin tolerance test showed that glucose tolerance and insulin sensitivity were reduced in MCSEKO mice. Glucose transporter 4 (GLU4) and PKG-1 expression levels and insulin receptor substrate-1(IRS1)/PI3K/Akt signaling pathway were downregulated whilst the mTOR/S6K/S6 pathway was enhanced in MCSEKO mice. These effects were reversed by the H_2_S supplement. Aerobic treadmill training significantly promoted glucose tolerance and insulin sensitivity and improved GLU4 and PKG-1 levels, promoted IRS1/PI3K/Akt signaling and suppressed mTOR/S6K/S6 signaling pathway in MCSEKO mice. Our data suggest that skeletal muscle CSE/H_2_S signaling is critical for the maintenance of insulin sensitivity, which is associated with maintaining the balance in PKG, PI3K/Akt, and mTOR/S6K/S6 signaling pathways in skeletal muscle.

## 1. Introduction

The skeletal muscle is the largest organ of the human body and accounts for 40–50% of the total body weight [1]. It is crucial for such important functions as exercise, respiration, and thermogenesis. In addition, skeletal muscle conducts a key role in maintaining circulating glucose homeostasis, because it is the largest insulin-sensitive organ, accounting for about 85% of the total body glucose intake.

Cystathionine-γ-lyase (CSE, EC 4.4.1.1) and cystathionine-β-synthetase (CBS, EC 4.2.1.22) are biologically essential and distributed in various tissues [2]. They independently synthesize hydrogen sulfide (H_2_S) via alternative desulfuration reactions in various tissues and catalyze the trans-sulfuration pathway. Under the catalysis of vitamin B6, CBS can condense homocysteine with serine to produce cystathionine. Cystathionine can then be hydrolyzed by CSE to form cysteine, which can be used to synthesize 

Antioxidant glutathione (GSH) or further metabolized into sulfate which is excreted in urine [3]. Both CBS and CSE proteins are expressed in human and rat skeletal muscles [4]. However, CSE but not CBS is identified in mouse skeletal muscles [5]. Ishii et al. [6] have demonstrated that global CSE^−/−^ mice display acute skeletal muscle atrophy and finally died of severe paralysis of the extremities when they are fed a diet with low cysteine, which implies that CSE plays an important role in skeletal muscle. Some studies have shown that H_2_S can modulate glucose transporter 4 (GLUT4) expression in cultured myoblast C2C12 cells. However, the physiological function of CSE in skeletal muscle remains to be elucidated.

Based on the above background, we sought to explore the roles of CSE/CBS/H_2_S signaling in skeletal muscle using a genetic modification approach. Since mouse skeletal muscle mainly expresses CSE, we generated skeletal muscle CSE knockout (MCSEKO) mice to examine the functional role of CSE in skeletal muscle. We found that the body weight, skeletal muscle morphology, and exercise capacity phenotypes were not affected in the MCSEKO mice. RNA-seq combined with a functional test indicated that the MCSEKO mice exhibited insulin resistance as evidenced by a decrease in intraperitoneal glucose tolerance test (IGTT) and insulin tolerance test (ITT) and down-regulated expression of insulin receptor substrate 1 (IRS1) and GLUT4. H_2_S donor treatment and aerobic treadmill exercise significantly ameliorated insulin resistance. Mechanistically, we demonstrated that CSE deficiency elicits a retrograde response in muscle to cause insulin signaling pathway IRS1/PI3K/Akt suppression and mammalian target of rapamycin (mTOR)/ribosomal protein S6 kinase (S6K)/S6 signaling pathway enhancement. Our data suggest that skeletal muscle CSE/H2S signaling is crucial for the maintenance of insulin sensitivity and circulatory glucose homeostasis.

## 2. Materials and Methods

### 2.1. Animals

CSE^Flox/Flox^ mice (NM-CKO-200274) and Ckmm-Cre (SJ-006475) were purchased from Shanghai model organisms (Shanghai, China). Mice were housed under a 12 h light/12 h dark cycle at 23 °C with free access to food and water. CSE^Flox/Flox^ mice were first mated with Ckmm-Cre mice. The MCSEKO mice were obtained by mating F1 CSE^Flox/+^; Cre^+^ mice to littermate CSE^Flox/+^; Cre^−^ mice. CSE^Flox/Flox^ mice were genotyped via polymerase chain reaction (PCR) using the following primers: 5′-GACAGTGAATCCTTTGGCAGTA-3′ and 5′- CACAGAGGGTAGATATT GAGACTTT-3′. The Ckmm-Cre mice were genotyped using the following primers: 5′-ATTTG CCTGCATT ACCGGTCG-3′ and 5′-CAGCATTGCTGTCACTTGGTC-3′. To avoid gender bias in our results, we used male mice in our experiments. All the procedures of animal studies were granted by the Ethics Review Committee for Animal Experimentation of the Shanghai University of Sport (approval number: 102772020DW008). 

### 2.2. Body Weight, Body Composition Measurement, and Skeletal Muscles Weight

The mice’s body weight and body composition were measured weekly from 3 weeks old to 20 weeks old. According to the manufacturer’s instructions, the EchoMRI body composition analyzer E26-310-M was used to noninvasively measure mice’s body composition. The mice were sacrificed with deep anesthesia at 21–22 weeks old, and the six sorts of skeletal muscles, namely quadriceps muscle (QUA), gastrocnemius muscle (GAS), triceps brachii (Triceps), tibialis anterior muscle (TA), extensor digitorum longus muscle (EDL), and soleus muscle (SOL) were dissected and weighted, then fixed with 4% paraformaldehyde or stored at −80 °C after snap freezing by liquid nitrogen. 

### 2.3. Treadmill Exhaustive Exercise Capacity and Grip Strength

The exhaustive capacity test was conducted according to prior studies [7]. Briefly, before the exercise test, the mice (22 weeks old) are subjected to adapt to the treadmill for 2 days. In adaptive exercise, the treadmill machine was set on a 15° incline. It started with a 5-min adaptation period of 0 m/min, then continued for 10 min at 10 m/min, followed by a 10-min movement at 14 m/min. In the exercise test, the machine was set on a 10° incline, started at a speed of 10 m/min, continued for 5 min, and increased 2 m/min every 5 min until the mice are exhausted (the mice stay on the electric shock for more than 5 s without resuming running) [7].

A grip strength meter (Columbus Instruments, Columbus, OH, USA) was used to evaluate the grip strength of mice. In each test, the mice were placed on the grip strength meter and pulled parallel to the ground until they lost grip. At each time point, the mice were given five tests, with a 10 s rest between tests. The average value was obtained from the best 3 out of the 5 tests. It was taken as the final score of each mouse.

### 2.4. Glucose and Insulin Tolerance Tests

When the mice were at 10, 15, and 20 weeks old, intraperitoneal glucose tolerance tests (IGTT) were performed. Before the IGTT test, the mice fasted overnight. Mice were intraperitoneally injected with glucose (1 g/kg) and their blood glucose level measured with a blood glucose meter (Accu-Chek Performa) at 0, 15, 30, 60, 90, and 120 min after injection. The insulin tolerance test (ITT) was performed on mice at 11, 16, and 21 weeks of age. For ITT, the mice fasted for 6 h before the experiment. Insulin (0.7 U/kg) was injected and blood glucose was measured 0, 15, 30, 60, 90, and 120 min after injection, using the average of each curve to calculate the incremental area under the curve (AUC). The mice were sacrificed at 21 weeks old with deep anesthesia, and muscle tissues were obtained, and then stored at −80 °C after snap freezing in liquid nitrogen.

### 2.5. Exogenous H_2_S Supplementation

The mice (14 weeks old) were intraperitoneally injected with GYY4137 (Sigma-Aldrich) at a dose of 50 mg/kg for 6 weeks. GYY4137 is a slow-releasing H_2_S donor and a water-soluble derivative of Lawesson’s reagent. It has been shown that GYY4137 can release H_2_S through hydrolysis [8]. The control group received the same volume of saline. After ITT and IGTT, the mice were sacrificed by deep anesthesia, and the muscle samples were collected. 

### 2.6. Aerobic Treadmill Exercise Protocol

The mice (14 weeks old) were subjected to aerobic treadmill exercise. In the first week, the mice were adapted to the machine. They were allowed to walk on the treadmill at a speed of 5 m/minute. After the adaptation period, the mice gradually carried out exercise training at the speed of 8–12 m/min, lasting for 60 min/day, 6 days/week, and 6 weeks. Concretely, the animals ran on the machine for 5 min (8 m/min) for warming up, then ran on the treadmill for 50 min (12 m/min), then ran for 5 min (8 m/min) for cooling down. The exercise intensity was moderate, about 60–75% of VO_2_ max [9,10,11]. The mice were subjected to ITT and IGTT, and then sacrificed by deep anesthesia, and the muscle samples were collected. 

### 2.7. HE Staining 

Skeletal muscle tissues were fixed overnight with 4% paraformaldehyde. The fixed tissue samples were then embedded in paraffin. A continuous cross-section of muscle tissues with a thickness of 5 μM was obtained, and hematoxylin eosin (HE) staining was performed using a commercial kit (KeyGEN, China). Stained slices were observed by using Olympus microscopy. The skeletal muscle cross-section area (CSA) was analyzed by using Image J software (version 1.8.0, Rawak Software Inc., Stuttgart, Germany).

### 2.8. Q-PCR

The total RNA of muscle tissues was extracted using the TRIzol (Invitrogen, Carlsbad, CA, USA) according to the manufacturer’s instructions. The concentration and quality of the extracted RNA were determined on a NanoDrop spectrophotometer (Thermo Scientific, Waltham, MA, USA). Next, 1 µg RNA was reverse transcribed into cDNA using a cDNA reverse transcription kit (Thermo Fisher). Q-PCR was conducted on QuantStudio 6 Flex real-time PCR system (Thermo Fisher). Next, 2 μL of diluted cDNA of various samples was mixed with SYBR Green PCR Master Mix (Life Technologies, Shanghai, China). The housekeeping gene *β-actin* was employed as an internal control. Primers were as followings: *CSE*, 5′-CGGGTTTTGAATACAGCC GC-3′(forward), 5′-CAGCAAGACCCGATGCAAAG-3′(reverse); *β-actin*, 5’-TGGAA GGTGGACAGTGAGGC-3’(forward), 5’-CCCAGGCATTGCTGACAGG-3’(reverse). At the end of amplification, the specificity of PCR products was detected by a melting curve. Subsequent sequencing of the PCR product was also performed to further confirm the specificity. To determine the relative quantification of gene expression of the target gene and housekeeping gene, compare Ct (threshold cycle) method and arithmetic formulate (2^−∆∆Ct^) was applied.

### 2.9. RNA-Seq Analysis

A total of six samples (3 GAS from MCSEKO mice and 3 GAS from Flox mice) were used for RNA-seq. NovoGene (Beijing, China) conducted all the procedures including RNA isolation, RNA quality evaluation (yield, purity, and integrity), construction of the cDNA library, and Illumina sequencing. In short, RNA sequence libraries were generated from mRNA purified from total RNA by using poly-T oligonucleotide-linked magnetic beads. The data was sequenced on Illumina NovaSeq 6000 using a 150 bp read run of paired reads. The data quality was checked on the Illumina Novaseq platform. Demultiplexing was conducted with the Illumina Bcl2fastq2 v 2.17 program. These readings were mapped to the latest UCSC transcript set using Hisat2 version 2.0.5, and gene expression levels were estimated using feature counts 1.5.0-p3. The trimmed mean of M-values (DESeq) was employed to normalize the gene expression. Differentially expressed genes were acquired using the DESeq2 R package (1.16.1), as defined by *p* < 0.05 and |log2FoldChange| > 0. Gene ontology (GO) enrichment analysis and Kyoto Encyclopedia of Genes and Genomes (KEGG) enrichment analysis were carried out for subsequent pathway analysis.

### 2.10. Western Blot Analysis

The GAS muscle was homogenized by an ultrasonic vibrator and mechanical homogenizer in a cold RIPA buffer containing a mixture of protease inhibitors (Beyotime, Nanjing, China). The tissue homogenates were centrifuged at 12,000× *g* for 15 min at 4 °C, and the supernatants were collected. The protein concentration was quantified by a BCA kit (ECOTOP, Guangzhou, China). The samples containing 30 μg protein were denatured, separated with 10% SDS-PAGE, and then transferred to the poly (vinylidene fluoride) membrane (Millipore Corp, Billerica, MA, USA) in turn. After being blocked, immunoblots were incubated with primary antibody anti-CSE (1:300; Santa Cruz Biotechnologies, California, USA), anti-GLUT4 (1:1000; Abcam, Cambridge, UK), anti- mTOR (1:1000; Cell Signaling Technology, Boston, MA, USA), anti-phospho-mTOR (p-mTOR) (1:1000; Cell Signaling Technology), anti-S6K (1:1000; servicebio), anti-phospho-S6K (p-S6K) (1:1000; absin, Shanghai, China), anti-ribosomal protein S6 (S6) (1:1000; servicebio, Wuhan, China), anti-phospho-S6 (p-S6) (1:1000; absin), anti-IRS1 (1:1000; servicebio), anti-phospho-IRS1 (p-IRS1) (1:1000; servicebio), anti-phosphoinositide 3-kinase (PI3K) (1:1000; servicebio), anti-phospho-phosphoinositide 3-kinase (p-PI3K) (1:1000; servicebio), anti-Akt (1:1000; servicebio), anti-phospho-Akt (p-Akt) (1:1000; servicebio), anti-PKG-1 (1:1000; Cell Signaling Technology), and anti-β-actin (1:10,000; Bioworld, Nanjing, China) at 4 °C overnight, and then incubate with IgG (1:5000; Abcam) conjugated with secondary horseradish peroxidase at room temperature for 1 h. Immunoreactive proteins were visualized using an enhanced chemiluminescence Western blotting detection system (Millipore). A densimeter (Syngen, Braintree, UK), Genesnap, and Genetools software (Syngen, Braintree, UK) were used to measure the staining intensity of the bands. To control sampling errors, the ratio of band intensities to β-actin was obtained to quantify the relative protein expression level. 

### 2.11. Statistical Analysis

All data were presented as mean ± standard error of the mean (SEM). The normal distribution was evaluated by a Shapiro-Wilk test. Statistical significance was determined according to sample distribution and variance uniformity. An unpaired two-tailed Student’s *t*-test or nonparametric test was performed for two group comparison. For more than two groups, one-way analysis of variance (ANOVA) followed by post-hoc multiple comparisons were performed for intergroup comparisons. *p* < 0.05 was considered statistically significant. 

## 3. Results

### 3.1. The MCSEKO Mice Show Normal Body Weight, Body Composition, Muscles Mass, and Musculature Morphology

**Figure 1:**
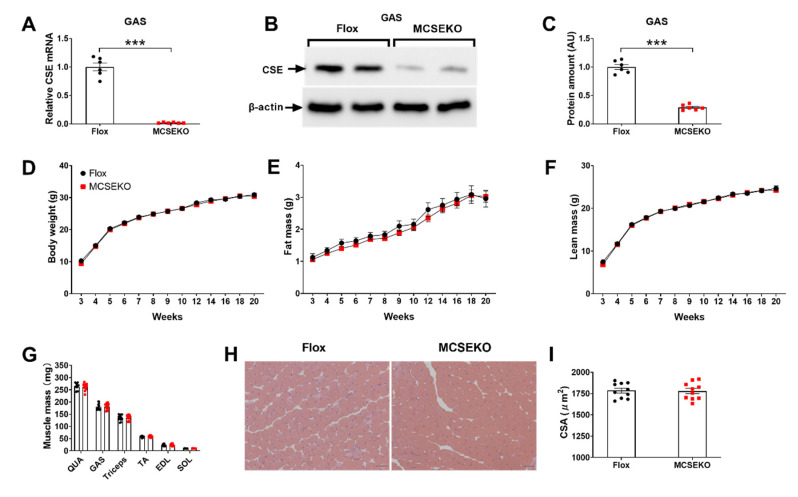
The expression level of CSE and characterization of body weight, body composition, muscle mass, and musculature morphology in MCSEKO mice. MCSEKO mice and littermate Flox mice were housed in standard conditions until 21-weeks-old, and then sacrificed under deep anesthesia. The body weight, body composition, and muscle mass were measured every 1 to 2 weeks as indicated. Q-PCR and Western blotting analysis were used to determine CSE expression in the skeletal muscle. (**A**) CSE mRNA levels in the GAS of Flox mice and MCSEKO mice (*n* = 6 in each group). (**B**,**C**) CSE protein expression in the GAS was detected by western blot analysis in the mice (*n* = 6 in each group). (**D**–**F**) The body weight and body composition were measured in Flox mice and the MCSEKO mice. The body weight (**D**), fat mass (**E**), and lean mass (**F**) of Flox mice and MCSEKO mice (*n* = 12 in each group). (**G**) The muscle weight of the mice (*n* = 12 in each group). (**H**) H&E staining of GAS (scale bars, 50 μm). (**I**) the GAS cross section area in the mice (*n* = 12 in each group). All data were expressed as means ± SEM. *** *p* < 0.001.

As shown in Figure 1A–C, the MCSEKO mice showed a significant decrease in mRNA and protein levels of CSE in skeletal muscle.

The MCSEKO mice displayed normal body weight, fat mass, and lean mass when compared with Flox littermates (Figure 1D–F). There was no significant difference in QUA, GAS, Triceps, TA, EDL, and SOL muscle mass between the MCSEKO mice and Flox littermates (Figure 1G). HE staining showed no obvious abnormity in MCSEKO mice (Figure 1H,I). 

### 3.2. The MCSEKO Mice Show Normal Grip Strength and Exercise Capacity

**Figure 2:**
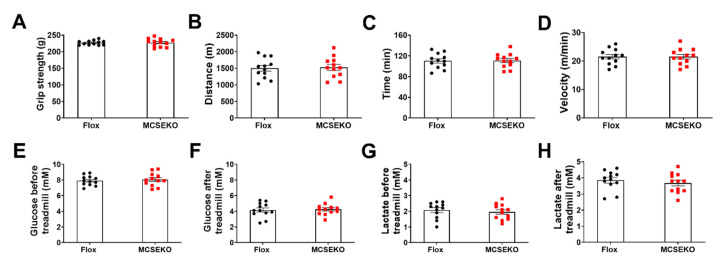
MCSEKO mice display normal grip strength and exercise capacity. The mice were housed under standard conditions. The grip strength and treadmill exhaustion exercise capacity were measured on Flox mice and the MCSEKO mice at 20 weeks old. (**A**) The grip strength of Flox mice and the MCSEKO mice (*n* = 12 in each group). (**B**–**D**) The running distance, running time, and running maximum velocity of Flox mice and the MCSEKO mice (*n* = 12 in each group). (**E**,**F**) The blood glucose level of Flox mice and the MCSEKO mice before and after running exhaustion (*n* = 12 in each group). (**G**,**H**) The blood lactate level of Flox mice and the MCSEKO mice before and after running exhaustion (*n* = 12 in each group). Data were expressed as means ± SEM.

As shown in Figure 2A–D, there was no significant difference in grip strength between MCSEKO mice and Flox littermates. Moreover, there was no significant significance in running distance, running time, and maximum running speed between MCSEKO mice and Flox littermates.

To test whether CSE deficiency affects exercise performance, the mice were subjected to a high-intensity treadmill. Before and after running, the blood glucose levels and blood lactate levels were measured to make sure that all mice reached the fatigue limit. As shown in Figure 2E–H, circulatory glucose level was decreased in MCSEKO and control mice after exercise, in contrast, the circulatory lactic acid level was increased after exercise in both of MCSEKO and control groups. There was no significant difference in blood glucose and lactic acid levels between the two groups before or after exercise. 

### 3.3. MCSEKO Mice Show Impaired Glucose Tolerance and Reduced Insulin Sensitivity with Age and GYY4137 Treatment Improved Them

**Figure 3:**
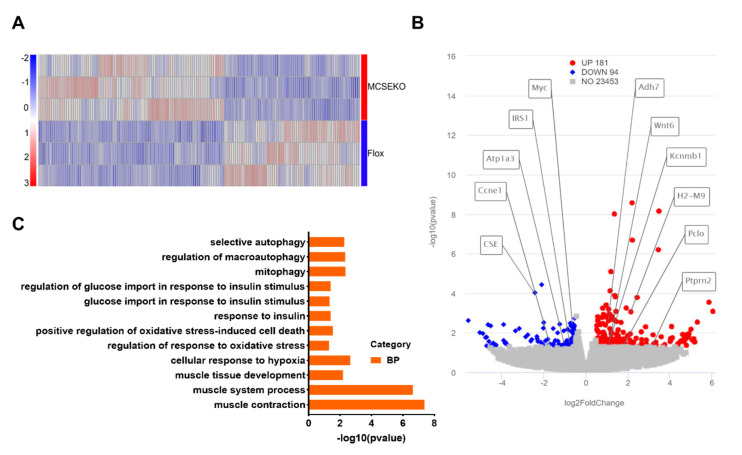
RNA-seq based transcriptome of skeletal muscle of MCSEKO mice. The mice were housed under standard conditions until 20-weeks-old, and then sacrificed under deep anesthesia. GAS was obtained for RNA-seq analysis. (**A**) Heat map of differentially expressed genes in the GAS of Flox mice and the MCSEKO mice (*n* = 3 in each group). (**B**) Volcano plot of differentially expressed genes in GAS of Flox mice and the MCSEKO mice (*p* < 0.05 and |log2FoldChange| > 0). The top eleven significantly different genes between the two groups were labeled. (**C**) The GO pathway enrichment analysis.

To study whether CSE deficiency induces molecular biological changes in skeletal muscle, a RNA-seq assay was performed on the GAS of the MCSEKO and Flox mice (Figure 3). The results showed that 275 genes were significantly differentially regulated. Among the differentially regulated genes, 181 genes were upregulated whilst 94 genes were down-regulated in CSE knockout mice compared with Flox mice (Figure 3A,B). GO analysis showed that selective autophagy, response to insulin, glucose import in response to insulin stimulus, and oxidative stress were enriched (Figure 3C). 

**Figure 4:**
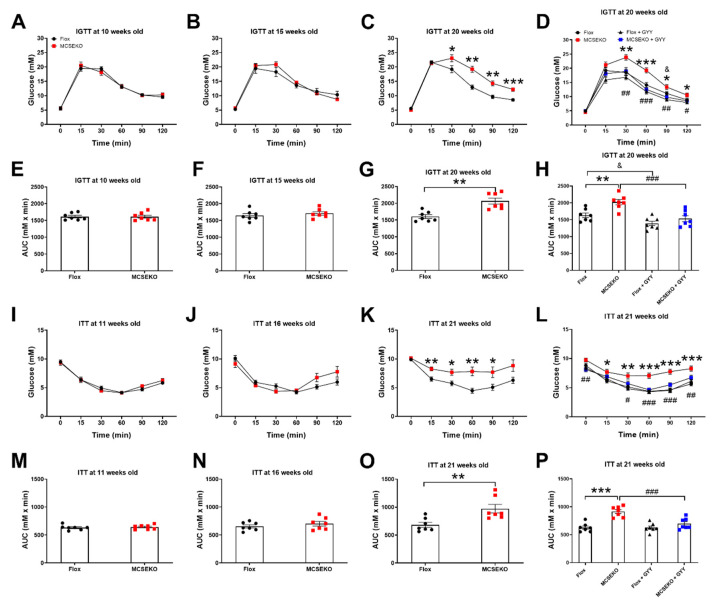
Glucose tolerance and insulin sensitivity are impaired in MCSEKO mice and GYY4137 treatment improves them. The mice were housed under standard conditions. The glucose tolerance tests in Flox mice and the MCSEKO mice at the age of 10 (**A**,**E**), 15 (**B**,**F**), and 20 (**C**,**G**) weeks, respectively (*n* = 7 in each group). The insulin tolerance tests in Flox mice and the MCSEKO mice at the age of 11 (**I**,**M**), 16 (**J**,**N**), and 21 (**K**,**O**) weeks, respectively (*n* = 7 in each group). Some mice at 14 weeks old were treated with GYY4137 for six weeks. (**D**,**H**) the glucose tolerance tests were performed at the age of 20 weeks (*n* = 7 in each group). (**L**,**P**) the insulin tolerance tests were performed at the age of 21 weeks (*n* = 7 in each group). Data were expressed as means ± SEM. * *p* < 0.05, ** *p* < 0.01, *** *p* < 0.001 (MCSEKO vs. Flox). # *p* < 0.05, ## *p* < 0.01, ### *p* < 0.001 (MCSEKO +GYY vs. MCSEKO). ^&^ *p* < 0.05 (MCSEKO +GYY vs. MCSEKO). GYY, GYY4137.

Next, we performed the IGTT and ITT to examine glucose tolerance and insulin sensitivity. The MCSEKO mice showed normal IGTT and ITT relative to Flox mice at 10~11 weeks old (Figure 4A,E,I,M) and 15~16 weeks old (Figure 4B,F,G,N). Impaired glucose tolerance and insulin tolerance occurred at 20-weeks-old. As shown in Figure 4C, circulatory glucose concentration was significantly increased 30, 60, 90, and 120 min after glucose administration in MCSEKO mice compared with Flox littermates. The area under the curve (AUC) of the blood glucose level was significantly higher in MCSEKO mice than that in Flox mice (Figure 4J). Moreover, ITT showed that significantly increased glucose levels occurred at 15, 30, 60, and, 90 min after insulin administration in MCSEKO mice compared with Flox littermates (Figure 4K). The AUC of blood glucose level was significantly higher in MCSEKO mice than that in Flox mice (Figure 4O). 

We then examined the effects of H_2_S donor GYY4137 treatment on glucose tolerance and insulin sensitivity in MCSEKO mice. With the six-week GYY4137 treatment, impaired glucose tolerance and reduced insulin sensitivity in MCSEKO mice were significantly reversed (Figure 4D,H,L,P). 

### 3.4. The Insulin Signal Pathway and GLUT4 Protein Level Are Down-Regulated in the Skeletal Muscle of the MCSEKO Mice and GYY4137 Treatment Ameliorates Them

**Figure 5:**
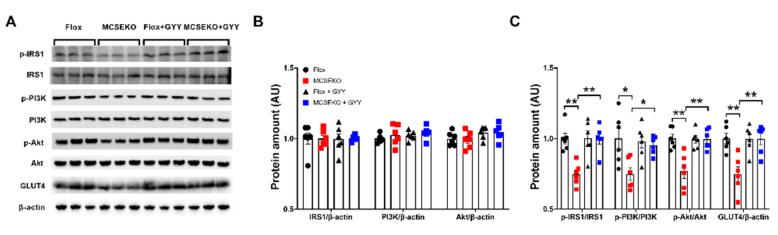
The skeletal muscle levels of IRS/PI3K/Akt signaling and GLUT4 are down-regulated in MCSEKO mice and GYY4137 treatment can increase them. MCSEKO mice and Flox mice were treated with GYY4137 or vehicle for six weeks. Western blot analysis of p-IRS1, IRS1, p-PI3K, PI3K, p-Akt, Akt, and GLUT4 protein levels in GAS muscle. (**A**) The representative images of western blotting. (**B**,**C**) Cumulative data of western blotting images. Data were expressed as means ± SEM (*n* = 6 in each group). * *p* < 0.05, ** *p* < 0.01.

In skeletal muscle cells, insulin promotes glucose uptake by binding to insulin receptors and subsequently activating a series of signal events triggered by IRS 1 phosphorylation. PI3K signaling interacts with p-IRS via p85 subunit, subsequently leads to Akt phosphorylated. This reaction leads to membrane translocation of GLUT4, thereby promoting glucose uptake [12]. It was found that the p-IRS1/IRS1, p-PI3K/PI3K, and p-Akt/Akt were down-regulated in MCSEKO mice compared to the littermate control mice (Figure 5). Six-week GYY4137 treatment increased p-IRS1/IRS1, p-PI3K/PI3K, and p-Akt/Akt levels compared with vehicle treatment in MCSEKO mice.

The predominant transporter for glucose uptake is GLUT4 in skeletal muscle. It was found that the GLUT4 expression was significantly decreased in the skeletal muscle of the MCSEKO mice compared to the littermate control mice (Figure 5). With six-weeks GYY4137 treatment, the GLUT4 protein levels in the skeletal muscle were significantly increased in MCSEKO mice.

### 3.5. The PKG-1 Protein Level Was Down-Regulated and the mTOR Signal Pathway Was Up-Regulated in the Skeletal Muscle of the MCSEKO Mice and GYY4137 Treatment Attenuated Them

**Figure 6:**
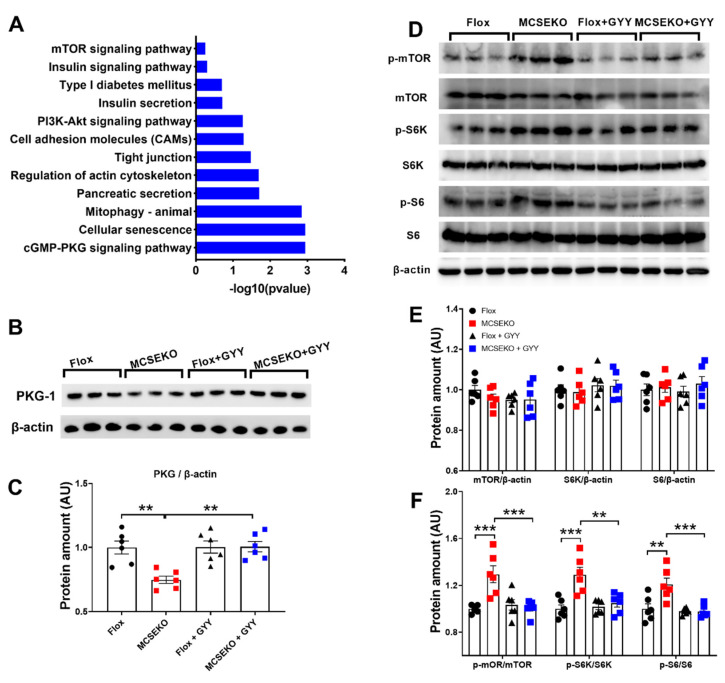
The PKG-1 level and mTOR signal pathway are altered in MCSEKO mice and GYY4137 treatment reverses them. MCSEKO mice and Flox mice were treated with GYY4137 or vehicle for six weeks. Western blot analysis of PKG-1 and mTOR signal pathway in GAS muscle. (**A**) Enriched pathways of KEGG analysis, (**B**) The representative images of western blotting of PKG1. (**C**) Cumulative data of western blotting images of PKG-1. (**D**) The representative images of western blotting of mTOR signaling pathway. (**E**,**F**) Cumulative data of western blotting images of mTOR signaling pathway. Data were expressed as means ± SEM (*n* = 6 in each group). ** *p* < 0.01,*** *p* < 0.001.

KEGG analysis showed that a series of signaling pathways including the cGMP-PKG, insulin signal, PI3K-Akt signal, and mTOR signal were enriched (Figure 6A). Some studies have shown that the CSE/H_2_S deficiency significantly decreased glucose metabolism in C2C12 myotubes and gastrocnemius muscle of high-fat diet-fed mice [13]. More recently, it is reported that the cGMP/PKG signaling is involved in glucose uptake [14]. As shown in Figure 6B,C, PKG-1 expression was significantly reduced in the skeletal muscle of MCSEKO mice compared to the littermate control mice. The PKG-1 protein level in MCSEKO mice was significantly increased after the GYY4137 treatment compared with the saline treatment. 

Insulin signaling is regulated by mTOR/S6K/S6 signaling pathway [15]. S6K, the downstream target gene of mTOR, can inhibit the tyrosine phosphorylation of IRS-1, thereby leading to impaired insulin signaling [16]. As shown in Figure 6D–F, MCSEKO mice showed significantly increased mTOR/S6K/S6 phosphorylation levels compared to the littermate control mice (Figure 6D–F). GYY4136 treatment significantly reduced mTOR/S6K/S6 phosphorylation levels compared with vehicle treatment in MCSEKO mice. 

### 3.6. Aerobic Treadmill Exercise Improved Glucose Tolerance and Insulin Resistance in the MCSEKO Mice

**Figure 7:**
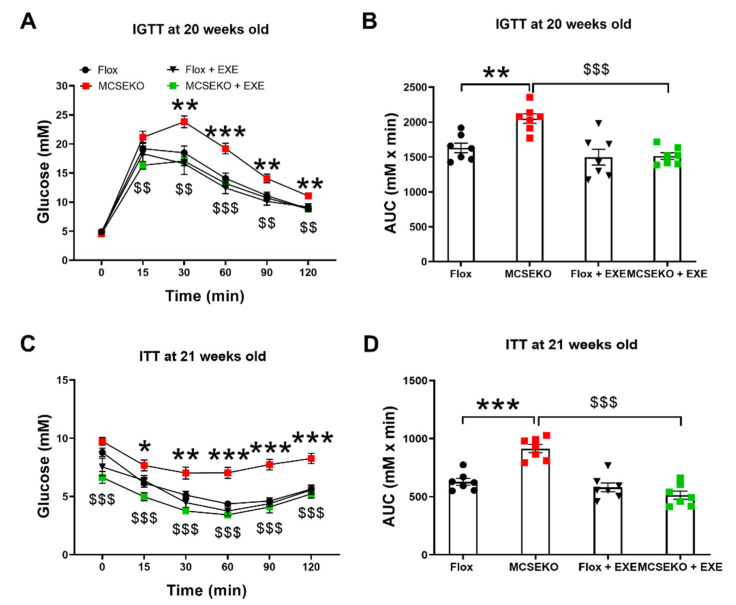
Aerobic treadmill exercise improves glucose tolerance and insulin sensitivity in MCSEKO mice. MCSEKO mice and Flox mice were housed under standard conditions. Aerobic treadmill exercise was performed on the mice at 14 weeks old for six weeks. (**A**,**B**) IGTT in Flox mice, the MCSEKO mice, Flox + EXE mice, and the MCSEKO + EXE mice at the age of 20 weeks (*n* = 7 in each group). (**C**,**D**) ITT in Flox mice, the MCSEKO mice, Flox + EXE mice, and the MCSEKO + EXE mice at the age of 21 weeks (*n* = 7 in each group). Data are expressed as means ± SEM. * *p* < 0.05, ** *p* < 0.01, *** *p* < 0.001 (Flox vs. MCSEKO); $$ *p* < 0.01, $$$ *p* < 0.001 (MCSEKO vs. MCSEKO + EXE). EXE: exercise.

Many studies have implicated that aerobic exercise can improve glucose tolerance and insulin resistance in obese [17] and diabetic mice [18]. Exercise enhances insulin action in the skeletal muscle [18,19]. We next tested whether aerobic treadmill exercise improved glucose tolerance and insulin resistance in MCSEKO mice. As shown in Figure 7, aerobic treadmill exercise significantly improved glucose tolerance performance and insulin sensitivity in MCSEKO mice.

### 3.7. Aerobic Treadmill Exercise Increases GLUT4 Expression and Improves PKG-1, IRSI/PI3K/Akt and mTOR/S6K/S6 Signaling Pathways in MCSEKO Mice

**Figure 8:**
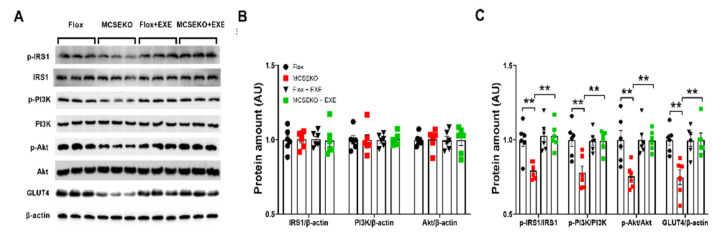
Aerobic treadmill exercise increases GLUT4 levels and IRSI/PI3K/Akt signaling in MCSEKO mice. MCSEKO mice and Flox mice were housed under standard conditions. Aerobic treadmill exercise was performed on the mice at 14 weeks old for six weeks. Western blot analysis of p-IRS1, IRS1, p-PI3K, PI3K, p-Akt, Akt, and GLUT4 levels in GAS. (**A**) The representative images of western blotting. (**B**,**C**) Cumulative data of western blotting images. Data were expressed as means ± SEM (*n* = 6 in each group). ** *p* < 0.01. EXE: exercise.

As shown in Figure 8, aerobic treadmill exercise increased GLUT4 protein levels and p-IRS1/IRS1, p-PI3K/PI3K, and p-Akt/Akt levels in MCSEKO mice compared with sedentary treatment. 

**Figure 9:**
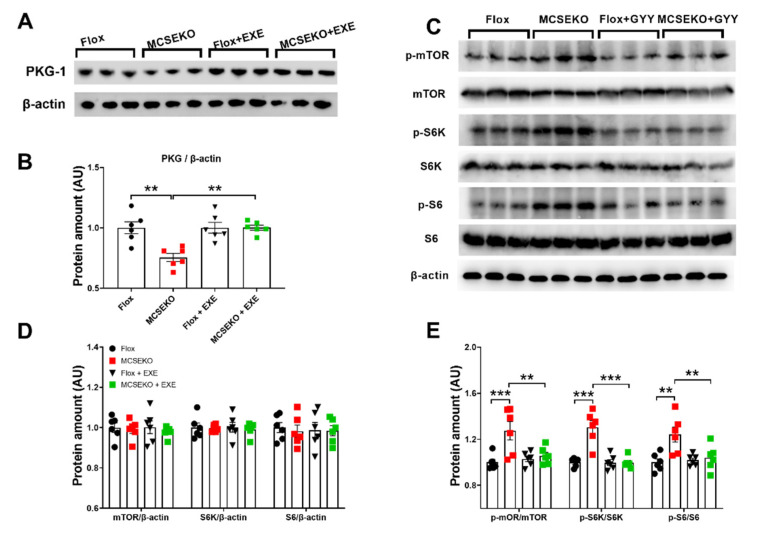
Aerobic treadmill exercise increases PKG-1 level and suppresses mTOR/S6K/S6 signaling in the skeletal muscle of the MCSEKO mice. MCSEKO mice and Flox mice were housed under standard conditions. Aerobic treadmill exercise was performed on the mice at 14-weeks-old for six weeks. (**A**,**B**) Western blot analysis of PKG-1 protein level in GAS muscle. (**C**–**E**) Western blot analysis of p-mTOR, mTOR, p-S6K, S6K, p-S6, S6 protein levels in GAS. (**A**,**C**) The representative images of western blotting. (**B**,**D**,**E**) Cumulative data of western blotting images. Data were expressed as means ± SEM (*n* = 6 in each group). ** *p* < 0.01, *** *p* < 0.001. EXE: exercise.

Figure 9 showed that the aerobic treadmill exercise increased PKG-1 protein level (Figure 9A,B) and decreased mTOR/S6K/S6 phosphorylation levels compared with sedentary treatment in MCSEKO mice. 

## 4. Discussion

Our study showed that CSE ablation in skeletal muscle led to glucose intolerance and insulin resistance, which was associated with decreased GLUT4 protein levels and imbalances in PKG-1, IRS/PI3K/Akt, and mTOR/S6K/S6 signaling pathways. 

Lu et al. [20]. have recently reported that high glucose, palmitate, and oleate levels significantly reduce H_2_S and the expression of CSE in mouse C2C12 myoblasts. The CSE expression level are declined in the skeletal muscle of db/db mice, which is reversed by the administration of NaHS [20]. Some other studies have also shown that GLUT4 expression is modulated by the CSE/H_2_S system in cultured muscle cells. The CSE knockdown leads to reduced GLUT4 expression and glucose uptake in C2C12 myotubes, which is prevented by H_2_S treatment [21]. *l*-cysteine, the precursor of H_2_S, promotes the translocation of GLUT4 to the cell membrane and promotes glucose uptake in C2C12 cells [22]. Cai et al. [23] revealed that treatment of high-fat diet-induced obese mice with GYY4137 improves insulin resistance. Manna et al. [24] observed that exogenous supplementation with Na_2_S (a source of H_2_S) up-regulates insulin-stimulated glucose utilization via up-regulating the insulin signaling pathway. Consistent with these studies, we also revealed that CSE/H_2_S is an important factor in maintaining insulin sensitivity and GLUT4 expression in skeletal muscle.

When insulin binds to the extra-cellular domain, the insulin receptor is activated through trans-autophosphorylation [25]. Afterward, the tyrosine site of IRS-1 undergoes phosphorylation, which binds to PI3K, enabling PI3K to activate Akt [26]. Xue and colleagues [27] have demonstrated that glucose uptake is decreased in cultured myotubes with CSE knockdown. Exogenous H_2_S treatment increases glucose uptake via activation of the IRS/PI3K/Akt signaling pathway in L6 myotubes. H_2_S treatment improves insulin sensitivity and increases glucose tolerance via promoting PI3K/Akt signaling in skeletal muscle in Goto-Kakizaki diabetic rats. In the current study, the muscle CSE deficiency led to glucose intolerance and poor insulin sensitivity, and reduced GLUT4 expression level and IRS/PI3K/Akt signaling pathway. GYY4137 treatment significantly increased glucose tolerance and insulin sensitivity and promoted IRS1/PI3K/Akt signaling and GLUT4 protein level in the skeletal muscle of the MCSEKO mice. Together, it suggests that CSE/H_2_S maintains insulin sensitivity via regulating IRS/PI3K/Akt signaling pathway in skeletal muscle. Of note, GYY4137 treatment has no effects on glucose tolerance and insulin resistance in wild type mice, which is consistent with the Manna et al.’s study where they show that H_2_S and *l*-cysteine did not affect glucose utilization in the cultured cells at normoglycemic conditions [24].

It is known that mTOR is a component of the negative feedback loops in insulin signaling. IRS-1 phosphoration is suppressed by mTOR/S6K signaling [28]. In fact, insulin sensitivity is under control by the balance between mTOR signaling and PI3K/Akt signaling. Koketsu et al.’s study has indicated that suppression of the mTOR/S6K pathway may enhance Akt phosphorylation, restore insulin sensitivity, and improve glucose tolerance [29]. It has also been shown that mTORC1 mediates phosphorylation and the stability of growth factor receptor binding protein 10 (Grb10), one of the mTORC1 substrates, leading to feedback suppression of PI3K and inhibition of insulin signaling [15]. In addition, chronic mTOR activation contributes to insulin resistance by inhibiting insulin signaling through an S6K-independent negative feedback loop [30]. S6K1 KO mice show enhanced insulin sensitivity [31]. Interestingly, the present study has demonstrated CSE/H_2_S deficiency led to increased mTOR signaling, in contrast, it caused a reduction of PI3K/Akt signaling. This key piece of evidence may explain the down-regulated insulin signaling observed in MCSEKO mice.

The cyclic GMP-PKG signaling pathway is involved in the modulation of glucose uptake in skeletal muscle [32]. The stimulating effects of cGMP on glucose transport require the activation of PKG in muscle cells [33]. PKG activators significantly increase glucose uptake in cultured rat podocytes [34]. Suppression of PKG signaling would inhibit insulin sensitivity [34,35]. The present study showed that CSE/H_2_S signaling maintains PKG-1 expression in skeletal muscle, which may indicate that the effects of CSE/H_2_S signaling on insulin sensitivity are associated with its regulating PKG-1 expression.

Oxidative stress is involved in the development of insulin resistance [36]. H_2_S suppresses mitochondrial oxidative stress in by increasing the intracellular GSH concentration [37]. Parsanathan et al. [21] reveal that H_2_S deficiency or CSE knockdown increases total protein S-glutathionylation, which protects proteins from oxidative stress. Exogenous H_2_S supplementation increases GSH levels, attenuates cellular oxidative stress, and improves glucose uptake in CSE knockdown myotubes. Manna et al. [24] show that H_2_S promoting glucose uptake is associated with increasing cellular GSH content, reduces oxidative stress, and activates IRS1/PI3K/AKT signaling in cultured adipocytes. Interestingly, we found that oxidative stress was enriched in GO analysis. In fact, CSE deficiency can result in oxidative stress since CSE is an upstream factor for GSH metabolism [3]. In addition, H_2_S can exert its effects by direct sulfhydration of various proteins. Cai et al. [23] demonstrated that CSE/H_2_S can sulfhydration of PPARγ at the C139 site and increase PPARγ activity, thereby increasing insulin sensitivity and promoting glucose uptake.

Exercise can enhance the expression of the GLUT4 glucose transporter [38]. After one exercise, the increase of GLUT4 transcription lasted for 3–24 h [39]. In addition, long-term regular exercise can increase the expression of GLUT4 protein steadily and improve blood glucose control [40]. It has been also reported that the expression of GLUT4 protein and glucose intake stimulated by insulin in skeletal muscles is increased after three weeks of single-leg standing training, compared with the untrained muscles [41]. Similarly, Christ-Roberts et al. stated that endurance training enhances the expression of GLUT4 in patients with diabetes and reduces circulatory glucose levels [42]. In fact, some studies have proposed that exercise promotes insulin signaling [43,44]. Similarly, in this study, we also observed that 6-week aerobic treadmill exercise training enhanced GLUT4 protein expression, increased the phosphorylation IRS1/PI3K/Akt protein levels, and improved the systematic insulin sensitivity and glucose tolerance in the MCSEKO mice.

Some studies have demonstrated that the benefit of aerobic exercise on insulin resistance is associated with the suppression of mTOR/S6K signaling pathway in skeletal muscle. Niu et al. [45] reported that aerobic exercise improves glucose tolerance and reduces mTOR/S6K signaling in skeletal muscle in high-fat diet mice. Glynn et al. [46] have also found that six-week aerobic exercise and nine-week independent running wheel ameliorate insulin resistance by decreasing mTOR/S6K signaling in skeletal muscle. Consistent with these studies, we found that the 6-week aerobic treadmill exercise improved insulin sensitivity and glucose tolerance and attenuated the mTOR/S6K signal pathway in the MCSEKO mice. The above evidence suggests that aerobic exercise contributes to the reduction in the mTOR/S6K signaling pathway.

Zhang et al. [47] report that insulin sensitivity is increased via the cGMP/PKG pathway after several hours of contraction of mouse skeletal muscle. Fan et al. [48]. reveal that PKG signaling can increase Hsp20 phosphorylation at Ser16. Boluyt et al. [19] demonstrated that aerobic exercise training enhances the Hsp20 level in the heart of rats, which can activate Akt signaling and improve insulin resistance [49]. Of note, we found that six-week aerobic treadmill exercise only improve the expression of PKG-1 in the muscle of the MCSEKO mice muscle but did not affect the expression of PKG-1 in the muscle of Flox mice muscle.

## 5. Conclusions

The present study revealed for the first time that skeletal muscle CSE/H_2_S deficiency leads to impaired glucose tolerance and insulin resistance in mice. Decreased GLUT4 expression and the imbalance in IRS-1/PI3K/Akt, PKG-1, and mTOR/S6K/S6 signaling pathways contribute to such effects. Exercise training ameliorates glucose intolerance and insulin resistance, and reverses the altered signaling pathways caused by CSE/H_2_S deficiency. Our study provides a novel mechanism regarding the roles of skeletal muscle in maintaining insulin sensitivity and blood glucose homeostasis, and benefits of exercise training in insulin sensitivity.

## Data Availability

The data presented in this study are available in the article.
